# Haploidentical transplants deliver equal outcomes to matched sibling transplants: a propensity score-matched analysis

**DOI:** 10.1186/s12967-023-04168-6

**Published:** 2023-05-18

**Authors:** Hengwei Wu, Yeqian Zhao, Fei Gao, Jimin Shi, Yi Luo, Jian Yu, Xiaoyu Lai, Lizhen Liu, Huarui Fu, Pengxu Qian, He Huang, Yanmin Zhao

**Affiliations:** 1 Bone Marrow Transplantation Center, The First Affiliated Hospital, Zhejiang University School of Medicine, No.79, Qingchun Road, Shangcheng District, Hangzhou, 310006 Zhejiang People’s Republic of China; 2 Institute of Hematology, Zhejiang University, Hangzhou, Zhejiang People’s Republic of China; 3 Zhejiang Province Engineering Laboratory for Stem Cell and Immunity Therapy, Hangzhou, Zhejiang People’s Republic of China; 4 Zhejiang Laboratory for Systems & Precision Medicine, Zhejiang University Medical Center, Hangzhou, Zhejiang People’s Republic of China

**Keywords:** Propensity score matching, Matched sibling donor, Haploidentical donor, Allogeneic hematopoietic stem cell transplantation

## Abstract

The success of allogeneic hematopoietic stem cell transplant for hematological malignancies is heavily dependent on the availability of suitable donors. Haploidentical donor (HID) and matched sibling donor (MSD) are two important donor options providing faster and easier sources of stem cells, however, due to confounding factors present in most retrospective studies, the validity of comparing outcomes between these two donor types remains uncertain. We conducted a post-hoc analysis of a prospective clinical trial (trial registration: Chinese Clinical Trial Registry; #ChiCTR-OCH-12002490; registered 22 February 2012; https://www.chictr.org.cn/showproj.aspx?proj=7061) to compare outcomes of HID versus MSD peripheral blood stem cell-derived transplants in patients with hematologic malignancies between 2015 and 2022. All HID-receiving patients had antithymocyte globulin-based conditioning. Propensity score matching was employed to minimize potential confounding factors between the two cohorts. A total of 1060 patients were initially reviewed and then 663 patients were ultimately included in the analysis after propensity score matching. The overall survival, relapse-free survival, non-relapse mortality rate and cumulative incidence of relapse were similar between HID and MSD cohorts. Subgroup analysis revealed that patients with positive measurable residual disease in first complete remission may have better overall survival with an HID transplant. The present demonstrated that haploidentical transplants can provide outcomes comparable to conventional MSD transplants, and HID should be recommended as one of the optimal donor choices for patients with positive measurable residual disease in first complete remission.

**Supplementary Information:**

The online version contains supplementary material available at 10.1186/s12967-023-04168-6.

## Introduction

Allogeneic hematopoietic stem cell transplantation (allo-HSCT) is a curative treatment for malignant hematologic diseases. The degree of human leukocyte antigen (HLA) compatibility between the donor and recipient is a critical factor in allo-HSCT success. Although a matched sibling donor (MSD) with the same HLA type is considered the optimal stem cell source, it is not available for up to 70–75% of patients [1]. Alternative options for patients without a suitable MSD include the use of matched unrelated donor, umbilical cord blood, and haploidentical donor (HID). While studies have demonstrated similar efficacy between matched unrelated donor and MSD transplant for treating malignant hematologic diseases, the likelihood of finding a matched unrelated donor for a patient is often low [2]. Although easily obtained, the low yield of hematopoietic stem cells restricts the usefulness of umbilical cord blood transplant in adult patients [3].

Due to the widespread use of HID, there is a global effort to determine the optimal donor option. The interaction between two immune systems with incompatible HLA presents potential risks, such as slower hematopoietic reconstitution, higher implantation failure, and an increased incidence of graft-versus-host disease (GVHD). However, incompatible HLA can also lead to graft versus leukemia effects from donor T and NK cells. Several studies have demonstrated that HID transplantation is similarly effective to MSD transplantation for treating malignant hematologic diseases [4, 5] yet other studies consistently demonstrate the superiority of MSD [6]. Due to the conflicting evidence, we aimed to compare the clinical outcomes of HID versus MSD transplants in two cohorts with hematologic malignancies from a prospective study. To ensure fair and unbiased assessments of the two predominant donor types in allo-HSCT, we conducted baseline matching. Propensity score matching analysis was used to rectify the initial imbalance between the HID and MSD cohorts. This approach allowed us to draw more credible and less biased conclusions from the less biased comparison between the two groups.

## Methods

### Study participants

This post hoc analysis of a prospective study which was registered at Chinese Clinical Trial Registry (#ChiCTR-OCH-12002490, URL: https://www.chictr.org.cn/showproj.aspx?proj=7061, date of registered: 02/22/2012) included 1060 patients with hematologic malignancies who underwent allo-HSCT from related donors at the First Affiliated Hospital of Zhejiang University between December 30, 2015, and January 6, 2022. Donor-recipient HLA typing from peripheral blood lymphocytes was performed by Zhejiang Provincial Blood Center or Shanghai Tissue Bank Co., Ltd using high-resolution mapping to evaluate six loci (HLA-A, -B, -C, -DRB1, -DQB1, and -DPB1). Patients were categorized into two cohorts based on receiving grafts from MSD or HID. The inclusion criteria were: (1) age > 8 years; (2) hematological malignancies such as acute myeloid leukemia, acute lymphocytic leukemia, myelodysplastic syndrome, myeloproliferative neoplasm, chronic myeloid leukemia and plasma cell leukemia; (3) receiving a first allo-HSCT; (4) peripheral blood stem cell transplantation from haploidentical relatives or matched sibling donors. The study adhered to the Declaration of Helsinki and was approved by the Ethics Review Committee of the First Affiliated Hospital of Zhejiang University School of Medicine.

### Transplant protocol

As previously described [7] all patients received either a myeloablative busulfan/cyclophosphamide-based conditioning regimen or a reduced intensity regimen consisting of fludarabine/busulfan. Antithymocyte globulin-Genzyme (ATG-G) or anti-thymocyte globulin Fresenius (ATG-F) was applied as preparation for haplo-HSCT. All patients received G-CSF mobilized peripheral blood stem cells and no graft was subjected to ex vivo T-cell depletion. GVHD prophylaxis consisted of cyclosporine, a short course of methotrexate, and mycophenolate.

### Propensity score matching analysis

Propensity score matching was conducted to mitigate selection bias and confounding factors by matching between the MSD and HID groups. The matching process incorporated patient age at transplant, sex, refined disease risk index (R-DRI), remission status at transplant, donor age, and sex, utilizing a fixed caliper width of 0.2. Furthermore, a 1:4 ratio was employed for matching the MSD group with the HID group.

### Endpoints and definitions

The study's primary objective was to evaluate 5-year overall survival (OS) after HSCT. Secondary endpoints included 5-year relapse-free survival (RFS), GVHD-free and relapse-free survival, cumulative incidence of relapse, cumulative non-relapse mortality (NRM), 100 day cumulative incidence of acute GVHD (aGVHD), and 5-year cumulative incidence of chronic GVHD (cGVHD) post-transplant.

The following definitions were used in this study: OS was defined as the period from transplant until the last follow-up or death from any cause. RFS was defined as the time from transplant until death, relapse, or last follow-up. GVHD-free and relapse-free survival was defined as the time from transplant until grade III-IV acute GVHD, severe chronic GVHD, relapse, or death. Relapse was defined as the reoccurrence of leukemia in previously achieved complete remission patients. Complete remission was defined as successful engraftment with 100% donor chimerism  < 5% leukemic cells in the bone marrow, and no leukemia cells in peripheral blood or extramedullary location. NRM was defined as death from any causes except underlying malignancy relapse/recurrence. aGVHD was defined and graded following the Mount Sinai Acute GVHD International Consortium consensus [8] while cGVHD was defined and graded following the National Institutes of Health criteria [9].

### Statistical analysis

Appropriate univariate comparisons of parameters were conducted using the χ2 test, Fisher’s exact test, student t-test, or Mann‒Whitney U test. Survival functions were estimated using the Kaplan–Meier method and differences were compared using the log-rank test. The cumulative incidence of relapse, NRM, engraftment, and GVHD were calculated cumulatively, with cumulative incidence of relapse and NRM estimated using the proportional hazards method. A Cox proportional hazard regression model was used for univariate and multivariate analyses of OS, RFS, GVHD-free and relapse-free survival, cumulative incidence of relapse, and NRM to assess the impact of HID on MSD in subgroups. Factors with* P* < 0.05 in univariate analyses were included in the final multivariate model. Hazard ratios (HR) with 95% confidence intervals (CI) were calculated. Statistical analysis was performed using SPSS statistical software version 22.0.01 (IBM, NY, USA) and R statistical software (version 3.4.3; http://www.r-project.org). A significance level of *P* < 0.05 (two-sided) was used.

## Results

### Patient characteristics

**Fig. 1:**
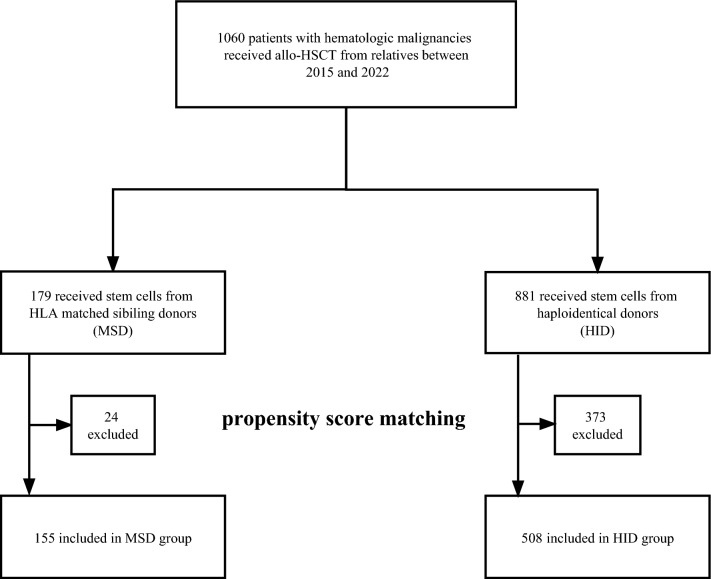
Study flow diagram. Diagram showing patients included in the final analysis. *Allo-HSCT* allogeneic hematopoietic stem cell transplant

In our center, a total of 1060 patients who received stem cells from related donors were enrolled in the study. Among them, 179 (15.2%) received unmanipulated MSD transplants, and 881 (74.7%) received unmanipulated HID transplants. To balance patient sex, D-DRI, remission status at transplant, and donor sex, we conducted propensity score matching analysis, resulting in the inclusion of 155 (23.4%) MSD and 508 (76.6%) HID transplant patients in the final analysis (Fig. 1). Among propensity-score matched populations, median (range) follow-up was 22.6 (0.3–66.4) and 20.9 (0.1–77) months for MSD and HID cohorts, respectively (*P* = 0.63). Median (range) age at HSCT was 39 years (11–58) and 36 (9–67) years for MSD and HID (*P* = 0.43), respectively. To be notice, in HID group 358 (70.5%) patients received ATG-G, while 150 (29.5%) patients received ATG-F. The type of ATG did not affect the OS of haplo-HSCT (*P* = 0.55, Additional file 1: Figure S1). Patient characteristics of the MSD and HID groups are summarized in Table 1, and post-transplant parameters are detailed in Additional file 1: Table S1.

**Table 1:** Characteristics of patients before and after propensity score matching analysis

	Unmanipulated data			Propensity score matching analysis		
	MSD	HID	*P* value	MSD	HID	*P* value
n (%)	179 (16.9)	881 (83.1)		155 (23.4)	508 (76.6)	
Patient sex			**0.005**			0.12
Male	76 (42.5)	476 (54.0)		66 (42.6)	253 (49.8)	
Female	103 (57.5)	405 (46.0)		89 (57.4)	255 (50.2)	
Median months from diagnosis to HSCT	6.3 (1.5–231.9)	6.9 (1.6–187.5)	**0.009**	6.1 (1.5–156.3)	6.6 (1.5–184.8)	0.03
Median age at HSCT (years)	41 (11–58)	39 (9–67)	0.51	39 (11–58)	36 (9–67)	0.43
Age at HSCT (years)			0.20			0.42
< 40	82 (45.8)	450 (51.1)		81 (52.3)	284 (55.9)	
≥ 40	97 (54.2)	431 (48.9)		74 (47.7)	224 (44.1)	
Diagnosis			0.58			0.81
Acute myeloid leukemia	83 (46.4)	418 (47.4)		71 (45.8)	228 (44.9)	
Myelodysplastic syndrome/ myeloproliferative neoplasm	25 (14.0)	95 (10.8)		20 (12.9)	58 (11.4)	
Chronic myeloid leukemia	8 (4.5)	28 (3.2)		7 (4.5)	16 (3.1)	
Acute lymphocytic leukemia	63 (35.2)	338 (38.4)		57 (36.8)	205 (40.4)	
Plasma cell leukemia	0 (0.0)	2 (0.2)		0 (0.0)	1 (0.2)	
Lineage			0.39			0.40
Lymphoblastic malignancies	63 (35.2)	340 (38.6)		57 (36.8)	206 (406)	
Myelogenous malignancies	116 (64.8)	541 (61.4)		98 (63.2)	302 (59.4)	
Refined disease risk index			**0.05**			0.32
Low	19 (10.6)	70 (7.9)		17 (11.0)	44 (8.7)	
Intermediate	134 (74.9)	602 (68.3)		113 (72.9)	348 (68.5)	
High	21 (11.7)	168 (19.1)		20 (12.9)	93 (18.3)	
Very high	5 (2.8)	41 (4.7)		5 (3.2)	23 (4.5)	
Remission status			**0.01**			0.34
First complete remission, MRD negative	125 (69.8)	565 (64.1)		94 (60.6)	313 (61.6)	
First complete remission, MRD positive	32 (17.9)	115(13.1)		39 (25.2)	99 (19.5)	
Second or third complete remission	11 (6.1)	100 (11.4)		11 (7.1)	47 (9.3)	
Active disease	11 (6.1)	101 (11.5)		11 (7.1)	49 (9.6)	
Body mass index at HSCT (kg/m^2^)			0.13			0.10
< 18.5	12 (6.7)	92 (10.4)		11 (7.1)	60 (11.8)	
≥ 18.5	167 (93.3)	789 (89.6)		144 (92.9)	448 (88.2)	
Conditioning regimen			0.16			0.14
Myeloablative	167 (93.3)	792 (89.9)		146 (94.2)	459 (90.4)	
Reduced intensity	12 (6.7)	89 (10.1)		9 (5.8)	49 (9.6)	
Donor sex			**0.007**			0.65
Male	94 (52.5)	557 (63.2)		85 (54.8)	289 (56.9)	
Female	85 (47.5)	324 (36.8)		70 (45.2)	219 (43.1)	
Median donor age (years)	40 (10–61)	32 (8–64)	** < 0.001**	38 (10–57)	34 (9–64)	0.04
Donor age (years)			** < 0.001**			0.26
< 40	88 (49.2)	594 (67.4)		88 (56.8)	314 (61.8)	
≥ 40	91 (50.8)	287 (32.6)		67 (43.2)	194 (38.2)	
Donor-recipient sex			**0.002**			0.35
Female to female	47 (26.3)	160 (18.2)		42 (27.1)	126 (24.8)	
Male to male	38 (21.2)	165 (18.7)		38 (24.5)	160 (31.5)	
Female to male	38 (21.2)	311 (35.3)		28 (18.1)	93 (18.3)	
Male to female	56 (31.3)	245 (27.8)		47 (30.3)	129 (25.4)	
ABO match, no. (%)			0.60			0.58
Matched	95 (53.1)	468 (53.1)		81 (52.3)	257 (50.6)	
Major mismatched	34 (19.0)	200 (22.7)		29 (18.7)	121 (23.8)	
Minor mismatched	40 (22.3)	166 (18.8)		35 (22.6)	102 (20.1)	
Bidirectional mismatch	10 (5.6)	47 (5.3)		10 (6.5)	28 (5.5)	
Median follow-up (days)	23.3 (0.3–67.4)	20.3 (0.1–115.8)	0.43	22.6 (0.3–66.4)	20.9 (0.1–77.0)	0.63

Bold indicates the values with *P* < 0.05

*HID* haploidentical donor, *HSCT* hematopoietic stem cell transplantation, *MRD* measurable residual disease, *MSD*, HLA-matched sibling donor

### Engraftment

**Fig. 2:**
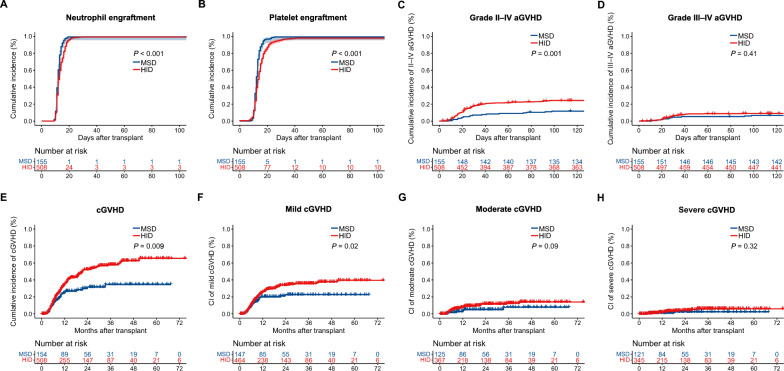
Cumulative incidence of neutrophil engraftment (**A**) platelet engraftment (**B**), grade II–IV aGVHD (**C**), grade III–IV aGVHD (**D**), overall cGVHD (**E**), mild cGVHD (**F**), moderate cGVHD (**G**), and severe cGVHD (**H**). *GVHD* graft-versus-host disease, *aGVHD* acute GVHD, *cGVHD* chronic GVHD

The median time for neutrophil engraftment was 12 days in the MSD group and 13 days in the HID group (*P* < 0.001, Fig. 2A). Platelet engraftment occurred at a median time of 12 days in the MSD group and 14 days in the HID group (*P* < 0.001, Fig. 2B). The cumulative incidence of neutrophil engraftment at day 30 was 99.4% (95% CI 95.5–99.9%) in the MSD group and 99.4% (95% CI 98.2–99.8%) in the HID group. The cumulative incidence of platelet engraftment at day 100 was 99.4% (95% CI 95.5–99.9%) in the MSD cohort and 98.0% (95% CI 87.9–96.1%) in the HID cohort.

### GVHD

The 100-day cumulative incidence of grades II–IV aGVHD was 11.7% (95% CI 6.5–16.6%) in MSD cohort and 24.3% (95% CI 20.5–28.0%; *P* = 0.001) in HID cohort (Fig. 2C). The incidence of grades III–IV aGVHD was 6.5% (95% CI 2.5–10.3%) and 8.6% (95% CI 6.1–11.0%; *P* = 0.41) in MSD and HID groups, respectively (Fig. 2C, D). Although the 5-year cumulative incidence rate of cGVHD was significantly higher in the HID group (48.1% [95% CI 41.3–54.1%] vs 29.2% [95% CI 19.9–37.5%], *P* = 0.009, Fig. 2E), the incidence of moderate to severe cGVHD did not differ between the two groups (Fig. 2G, H). However, a significant difference in the occurrence of mild cGVHD was observed (22.6% [95% CI, 14.6%-29.9%] vs 39.6% [95% CI 32.6–45.8%], *P* = 0.02, Fig. 2F).

### NRM and relapse

**Fig. 3:**
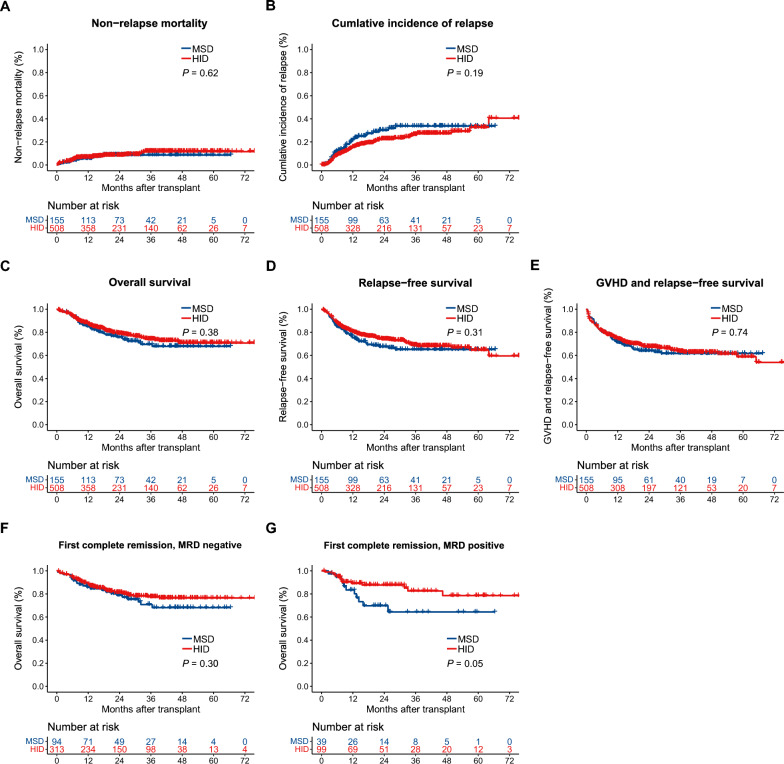
Outcome of HLA-matched sibling donor (MSD) and haploidentical donor (HID) groups. Cumulative incidence of non-relapse mortality **A**, and relapse (**B**). Probabilities of overall survival (**C**), relapse-free survival (**D**), and GVHD-free and relapse-free survival (E). Overall survival of patients in first complete remission with negative MRD (**F**) and positive MRD (**G**) at transplant. *GVHD* graft-versus-host disease, *MRD* measurable residual disease

The 5-year NRM rates in MSD and HID were similar, with values of 8.2% (95% CI 3.0–12.8) and 11.0% (95% CI 7.5–14.3%), respectively (*P* = 0.62, Fig. 3A). Additionally, there was no significant difference in the 5-year cumulative incidence of relapse between the two cohorts, with incidences of 28.6% (20.0–36.3%) for MSD and 27.8% (20.4–34.5) for HID (*P* = 0.19, Fig. 3B).

### OS, RFS and GVHD-free and relapse-free survival

A 5-year OS was comparable between twogroups [HR = 0.847 (MSD as reference), 95%CI 0.587–1.223, *P* = 0.38, Fig. 3C], with rates of 67.8% (95% CI 59.4–77.4%) in the MSD cohort and 70.7% (95% CI 65.5–76.4%) in the HID cohort. Similarly, the 5-year RFS was not significantly differentaccounting for 65.1% (95% CI 57.3–73.9%) in the MSD and 64.6% (95% CI 58.1–71.7%) in the HID (*P* = 0.31, Fig. 3D). The 5-year probability of GVHD-free and relapse-free survival for patients in the HID cohort was 53.9% (95% CI 52.1–66.4%), which was similar to MSD cohort (61.6%; 95%CI%, 53.8–70.6%, *P* = 0.74, Fig. 3E).

### Subgroup analyses

**Fig. 4:**
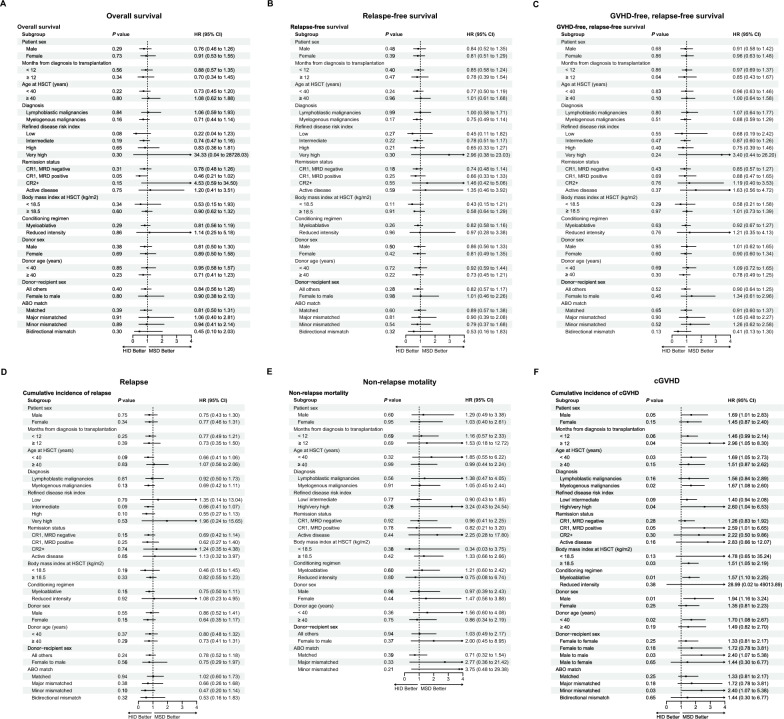
Forest plots for HRs and 95%CI of transplant outcomes in subgroup analyses. **A** Overall survival. **B** Relapse-free survival. **C** GVHD-free and relapse-free survival. **D** Cumulative incidences of relapse. **E** Cumulative incidence of non-relapse mortality. **F** Cumulative incidence of chronic GVHD. *GVHD* graft-versus-host disease; *MRD* measurable residual disease

We conducted subgroup analyses to identify patients who might benefit from different donors. Forest plots in Fig. 4A presented HRs for OS in subgroups. Notably, although patients in first complete remission and negative measurable residual disease (MRD) had similar OS in the HID and MSD cohorts (*P* = 0.30, Fig. 3F), patients in first complete remission and positive MRD at transplant showed a potentially better OS with HID (HR = 0.46, 95% CI 0.21–1.02, *P* = 0.05, Fig. 3G), However, no better RFS in the HID group for patients in first complete remission and MRD positivity (HR = 0.66, 95% CI 0.33–1.33, *P* = 0.25, Fig. 4B). No significant differences were observed in GVHD-free and relapse-free survival (Fig. 4C) and NRM (Fig. 4E) between the two cohorts. Whereas, analysis of cumulative incidence of relapse revealed a potential trend towards lower relapse risk in patients under 40 years of age (HR = 0.66, 95% CI 0.41–1.06, *P* = 0.01) and those with intermediate-risk R-DRI (HR = 0.66, 95% CI 0.41–1.07, *P* = 0.09) who received grafts from HID (Fig. 4D). The cumulative incidence of cGVHD was higher in the HID group than in the MSD group, as previously mentioned. But this trend did not consistently hold true for all subgroups. Notably, when either the patient or the donor was female, no increased incidence of cGVHD was observed in the HID group (Fig. 4F). Analysis of female patients (HR = 1.45, 95% CI 0.87–2.40, *P* = 0.15) and female donors (HR = 1.35, 95% CI 0.81–2.23, *P* = 0.25) revealed comparable rates of cGVHD between HID and MSD groups. We did not observe a significant increase in the incidence of cGVHD in the HID group for patients with lymphoblastic malignancies (HR = 1.56, 95% CI 0.84–2.89, *P* = 0.16), those aged ≥ 40 years at transplant (HR = 1.51, 95% CI 0.87–2.62, *P* = 0.14), older donors (≥ 40 years) (HR = 4.78, 95% CI 0.65–35.24, *P* = 0.12), or those with underweight status (BMI < 18.5 kg/m^2^) (HR = 1.49, 95% CI 0.82–2.70, *P* = 0.19).

### Multivariate analysis of main outcomes and contributing factors

No discernible variations in NRM, RFS, or OS were observed between the two groups, as determined by multivariate analysis (Table 2). The independent factors associated with worse RFS and OS were high/very high-risk R-DRI and grade III-IV aGVHD, while mild to moderate cGVHD was identified as an independent prognostic factor for better RFS and OS. Recipient age (≥ 40 years) was found to be an adverse factor for OS (HR = 1.511, 95%CI 1.059–2.156, *P* = 0.02) and NRM (HR = 2.160, 95%CI, 1.199–3.892, *P* = 0.01), but had no significant impact on RFS. Mild cGVHD was identified as an independent protective factor for NRM (HR = 0.325, 95%CI 0.137–0.772, *P* = 0.01), while grade III-IV aGVHD and severe cGVHD were independently associated with a higher probability of NRM (HR = 6.679, 95%CI 3.560–12.530, *P* < 0.001) and (HR = 3.860, 95%CI 1.343–11.097, *P* = 0.01), respectively.

**Table 2:** Multivariate analysis of survival and contributing factors

Outcomes	Hazard ratio (95% CI)	*P* value
**OS**		
Matched sibling donor	reference	0.45
Haploidentical donor	0.867 (0.597–1.260)	
Other significant factors		
Age at HSCT (years)		
< 40	Reference	**0.02**
≥ 40	1.511 (1.059–2.156)	
Refined disease risk index		
Low-intermediate	Reference	** < 0.001**
High-very high	2.176 (1.419–3.335)	
Remission status		
First complete remission, MRD negative	Reference	0.67
First complete remission, MRD positive	0.844 (0.536–1.328)	0.46
Second or third complete remission	0.969 (0.544–1.727)	0.92
Active disease	1.270 (0.716–2.251)	0.41
Acute GVHD		
0–II	Reference	** < 0.001**
III–IV	2.810 (1.732–4.559)	
Chronic GVHD		
None	Reference	** < 0.001**
Mild	0.360 (0.220–0.588)	** < 0.001**
Moderate	0.404 (0.176–0.929)	**0.03**
Severe	1.388 (0.606–3.181)	0.44
**RFS**		
Matched sibling donor	Reference	0.21
Haploidentical donor	0.808 (0.578–1.130)	
Other significant factors		
Refined disease risk index		
Low-intermediate	Reference	** < 0.001**
High-very high	2.227 (1.530–3.242)	
Remission status		
First complete remission, MRD negative	Reference	0.71
First complete remission, MRD positive	1.066 (0.723–1.572)	0.75
Second or third complete remission	1.148 (0.688–1.917)	0.60
Active disease	1.342 (0.811–2.219)	0.25
Acute GVHD		
0–II	Reference	**0.004**
III–IV	1.985 (1.252–3.148)	
Chronic GVHD		
None	Reference	**0.003**
Mild	0.532 (0.360–0.788)	**0.002**
Moderate	0.555 (0.280–1.099)	0.09
Severe	1.524 (0.709–3.277)	0.28
**NRM**		
Matched sibling donor	Reference	0.58
Haploidentical donor	1.211 (0.618–2.736)	
Other significant factors		
Age at HSCT (years)		**0.01**
< 40	Reference	
≥ 40	2.160 (1.199–3.892)	
Refined disease risk index		
Low-intermediate	Reference	0.35
High-very high	1.476 (0.658–3.310)	
Remission status		
First complete remission, MRD negative	Reference	0.77
First complete remission, MRD positive	0.831 (0.400–1.726)	0.62
Second or third complete remission	0.708 (0.241–2.079)	0.53
Active disease	1.312 (0.488–3.524)	0.59
Acute GVHD		
0–II	Reference	** < 0.001**
III–IV	6.679 (3.560–12.530)	
Chronic GVHD		
None	Reference	**0.001**
Mild	0.325 (0.137–0.772)	**0.01**
Moderate	0.347 (0.082–1.473)	0.15
Severe	3.860 (1.343–11.097)	**0.01**

Bold indicates the values with *P* < 0.05

## Discussion

Retrospective studies comparing MSD and HID are often subject to substantial statistical bias due to their non-randomized nature. Propensity score matching analysis can help to reduce variations across groups and improve the credibility of the study. In this prospective randomized study, we followed a standard transplant protocol, which allowed us to mimic a randomized controlled trial. We compared clinical data from recipients of malignant hematologic diseases in our center since 2015, addressing the most popular topic of transplant donor selection. The field of hematopoietic stem cell transplantation is rapidly advancing, with the implementation of modalities such as post-transplantation cyclophosphamide (PT/Cy). Since 2006, the efficacy of ATG-based haploidentical hematopoietic stem cell transplantation for treating malignant hematologic diseases was first reported. This prompted the initiation of China's ATG-based GVHD prevention program, which has been ongoing for nearly two decades [10]. The haplo-HSCT provides more transplant options for patients who lack matched and readily available donors. However, retrospective studies have reported that outcomes in terms of OS are better with MSD compared to HID [11, 12]. A previous five-year study was conducted from 2008 to 2013 in our center on patients undergoing allo-HSCT, comparing the advantages and disadvantages of HID versus MSD. The results indicated that MSD transplant conferred a superior OS compared to HID transplant [13]. Our latest findings suggest that ATG-based haplo-HSCT achieved comparable outcomes with MSD transplants.

The recurrence rate of HID transplants compared to MSD transplants is a subject of debate. A study of 10,679 acute leukemia patients found no significant difference in the likelihood of relapse between HID and MSD groups [14]. In a study of lymphoblastic and myelogenous malignancies, no significant difference was observed in the cumulative incidence of relapse between MSD and HID groups. Both groups exhibited a relatively high probability of relapse, with rates of 34% and 33%, respectively [4]. In a study of 189 patients in first complete remission, HID showed superior performance compared to MSD in high-risk acute myeloid leukemia, as indicated by improved RFS and a lower incidence of positive MRD flare after transplant [15]. In this present study, equivalent cumulative incidences of relapse were presented in two groups. Since the bias was delicately balanced, formulating sound results between groups. Analyses suggested that patients in first complete remission but MRD positivity could benefit more from haplo-HSCT regarding OS, indicating that HID grafts bear a stronger graft versus leukemia effect. Recent research from the Acute Leukemia Working Party of the European Society for Blood and Marrow Transplantation supported the notion that HID has more potent graft versus leukemia effects because the 2-year cumulative incidence of relapse was significantly lower in acute lymphoblastic leukemia patients who received HID compared to MSD. [16]

Older patients have a lower probability of finding a compatible MSD than younger patients because their siblings are sometimes also older and may not be physically able to donate. Donor age ≤ 45 years is associated with superior outcomes despite recipients' age [17, 18]. A fresh analysis conducted by the Chronic Malignancies Working Party of European Society for Blood and Marrow Transplantation suggested that the MSD remains the preferred choice over the HID in myelodysplastic syndromes, despite the baseline older donor age of 55 years in MSD than 36 years in HID [19]. A single-center study with significant inequivalent donor age showed similar outcomes achieved in HID and MSD groups [20]. Large variations of outcomes from several studies can be brought on because of baseline imbalances. Since the baseline data in the two cohorts were subtly balanced, we found that HID and MSD transplants had similar results in terms of the donor's age and that both options can have similar prognoses when given by donors of comparable ages. However, the donors in HID are composed of various kinships, resulting in a confounder to donor age. Notably, a study has demonstrated superior outcomes when using child donors compared to parental donors [18].

The occurrence of aGVHD in HID cohort was substantially higher compared to MSD group, whilst mostly grade I–II aGVHD. With an incidence of up to 50%, cGVHD is the most prevalent long-term complication of haplo-HSCT and one of the major factors that affect patients' long-term post-transplant quality of life [21]. Interestingly, as mentioned above, the cumulative incidence of cGVHD in the HID group was 1.5 times higher than that in the MSD group, especially in subgroups of male patients and/or male donors, myelogenous malignancies, and non-underweight status at transplant. Older patients and/or donors have no impact on the likelihood of developing cGVHD, hence both HID and MSD are feasible options in this regard. The incidence of cGVHD was higher in the HID group than in the MSD group for patients in first complete remission but MRD-positivity, supporting the earlier claim of increased OS with HID transplantation in this cohort. A significantly higher incidence of mild cGVHD was observed in haplo-HSCT, while moderate to severe cGVHD was no different from MSD, leading to a comparable impact on quality of life. NRM was not worse in the HID cohort, partially due to therapeutic supports and effective GVHD prophylaxis/therapies which have reduced transplant-associated mortality.

This study has several limitations. Firstly, although propensity score matching analysis can provide the study with attributes of a randomized controlled trial, it was a single-center study with inherent biases not fully balanced. Therefore, the conclusions may only apply to those who received ATG-based HID. Secondly, some critical cytogenetic characteristics were missing and perhaps not uniform between the two groups. Lastly, current studies suggest that patients over 40 years of age who receive an MSD transplant may require ATG for GVHD prophylaxis [22, 23]. We did not apply such preventive protocol until data analysis, which might underestimate the survival benefit of MSD transplant for patients > 40 years.

## Conclusions

Only a few patients, particularly in China, have an appropriate HLA identical sibling. For patients without an MSD, alternative sources of stem cells must be used immediately, with HID being the most common option. The long-term clinical outcomes were largely equivalent in the MSD and those with HID, however, in contrast to MSD, patients in first complete remission but positive MRD at transplant may benefit more from HID in terms of survival.

## Supplementary Information

**Additional file 1: Figure S1.** Probabilities of overall survival in haploidentical donor group regarding antithymocyte globulin type**.**

**Additional file 2: Table S1.** Post-transplant parameters of HLA-matched sibling donorand haploidentical donor.

## Data Availability

The datasets used or analyzed during the current study are available from the corresponding author on reasonable request.

## References

[1] Gragert L (2014). HLA match likelihoods for hematopoietic stem-cell grafts in the U S registry. N Engl J Med.

[2] Lv M, Huang XJ (2012). Allogeneic hematopoietic stem cell transplantation in China: where we are and where to go. J Hematol Oncol.

[3] Ballen KK, Gluckman E, Broxmeyer HE (2013). Umbilical cord blood transplantation: the first 25 years and beyond. Blood.

[4] Bashey A (2013). T-cell-replete HLA-haploidentical hematopoietic transplantation for hematologic malignancies using post-transplantation cyclophosphamide results in outcomes equivalent to those of contemporaneous HLA-matched related and unrelated donor transplantation. J Clin Oncol.

[5] Wang Y (2013). Long-term follow-up of haploidentical hematopoietic stem cell transplantation without in vitro T cell depletion for the treatment of leukemia: nine years of experience at a single center. Cancer.

[6] Inamoto Y (2019). Alternative donors: a match for matched sibling donors?. Lancet Haematol.

[7] Wu H (2022). Assessment of patient-specific human leukocyte antigen genomic loss at relapse after antithymocyte globulin-based t-cell-replete haploidentical hematopoietic stem cell transplant. JAMA Netw Open.

[8] Harris AC (2016). International, multicenter standardization of acute graft-versus-host disease clinical data collection: a report from the mount sinai acute gvhd international consortium. Biol Blood Marrow Transpl.

[9] Jagasia MH (2015). National Institutes of Health consensus development project on criteria for clinical trials in chronic graft-versus-host disease i the 2014 diagnosis and staging working group report. Biol Blood Marrow Transpl.

[10] Lu DP (2006). Conditioning including antithymocyte globulin followed by unmanipulated HLA-mismatched/haploidentical blood and marrow transplantation can achieve comparable outcomes with HLA-identical sibling transplantation. Blood.

[11] Bashey A (2016). comparison of outcomes of hematopoietic cell transplants from t-replete haploidentical donors using post-transplantation cyclophosphamide with 10 of 10 HLA-A, -B, -C, -DRB1, and -DQB1 allele-matched unrelated donors and hla-identical sibling donors: a multivariable analysis including disease risk index. Biol Blood Marrow Transpl.

[12] Salvatore D (2018). Outcomes of hematopoietic stem cell transplantation from unmanipulated haploidentical versus matched sibling donor in patients with acute myeloid leukemia in first complete remission with intermediate or high-risk cytogenetics: a study from the acute leukemia working party of the european society for blood and marrow transplantation. Haematologica.

[13] Luo Y (2014). T-cell-replete haploidentical HSCT with low-dose anti-T-lymphocyte globulin compared with matched sibling HSCT and unrelated HSCT. Blood.

[14] Ringdén O (2016). Is there a stronger graft-versus-leukemia effect using HLA-haploidentical donors compared with HLA-identical siblings?. Leukemia.

[15] Yu S (2020). Haploidentical transplantation might have superior graft-versus-leukemia effect than HLA-matched sibling transplantation for high-risk acute myeloid leukemia in first complete remission: a prospective multicentre cohort study. Leukemia.

[16] Nagler A (2021). Outcome of haploidentical versus matched sibling donors in hematopoietic stem cell transplantation for adult patients with acute lymphoblastic leukemia: a study from the acute leukemia working party of the european society for blood and marrow transplantation. J Hematol Oncol.

[17] Mehta J (2006). Does younger donor age affect the outcome of reduced-intensity allogeneic hematopoietic stem cell transplantation for hematologic malignancies beneficially?. Bone Marrow Transpl.

[18] DeZern AE (2021). Relationship of donor age and relationship to outcomes of haploidentical transplantation with posttransplant cyclophosphamide. Blood Adv.

[19] Raj K (2022). Comparison of outcomes for HLA-matched sibling and haplo-identical donors in Myelodysplastic syndromes: report from the chronic malignancies working party of EBMT. Blood Cancer J.

[20] Huang J (2020). Haploidentical related donor vs matched sibling donor allogeneic hematopoietic stem cell transplantation for acute myeloid leukemia and myelodysplastic syndrome aged over 50 years: a single-center retrospective study. Cancer Med.

[21] Lee SJ (2017). Classification systems for chronic graft-versus-host disease. Blood.

[22] Chang Y-J (2020). Antithymocyte globulin for matched sibling donor transplantation in patients with hematologic malignancies: a multicenter, open-label, randomized controlled study. J Clin Oncol.

[23] Zhang X-H (2021). The consensus from The Chinese society of hematology on indications, conditioning regimens and donor selection for allogeneic hematopoietic stem cell transplantation: 2021 update. J Hematol Oncol.

