# Supplementary figures and images for: Haploidentical transplants deliver equal outcomes to matched sibling transplants: a propensity score-matched analysis
Source: J Transl Med. 2023 May 18;21:329. doi: 10.1186/s12967-023-04168-6 (PMC10193779; doi:10.1186/s12967-023-04168-6)

# Haplo-HSCT

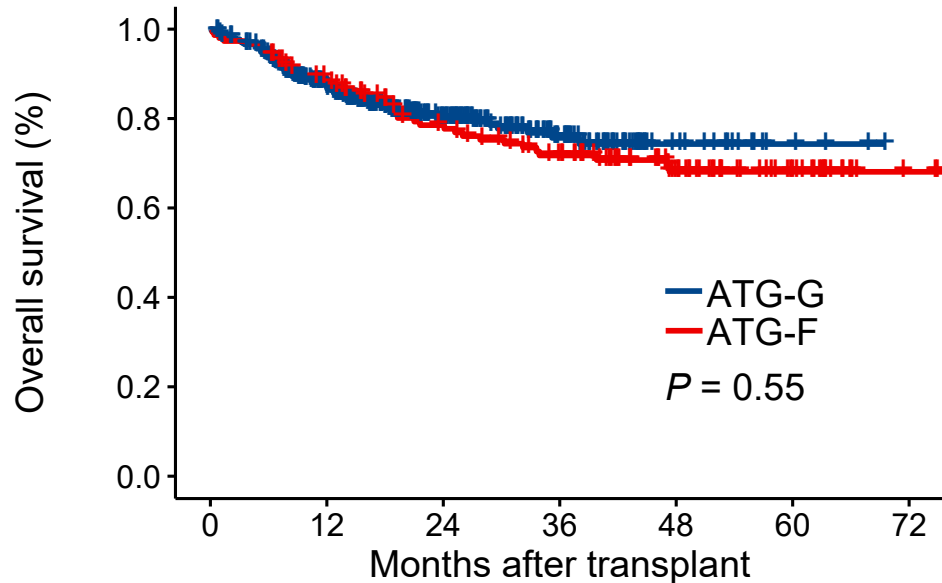

## Number at risk

|       |     |     |     |    |    |    |    |
|-------|-----|-----|-----|----|----|----|----|
| ATG-G | 358 | 239 | 137 | 64 | 18 | 4  | 0  |
| ATG-F | 150 | 126 | 97  | 79 | 47 | 25 | 7  |
|       | 0   | 12  | 24  | 36 | 48 | 60 | 72 |

Supplement: Supplementary file 1 — Additional file 1: Figure S1. Probabilities of overall survival in haploidentical donor group regarding antithymocyte globulin type. [file 12967_2023_4168_MOESM1_ESM.pdf]
