# Supplementary material for: Haploidentical transplants deliver equal outcomes to matched sibling transplants: a propensity score-matched analysis
Source: J Transl Med. 2023 May 18;21:329. doi: 10.1186/s12967-023-04168-6 (PMC10193779; doi:10.1186/s12967-023-04168-6)
Supplement: Supplementary file 2 — Additional file 2: Table S1. Post-transplant parameters of HLA-matched sibling donorand haploidentical donor. [file 12967_2023_4168_MOESM2_ESM.docx]

**Table S1. Post-transplant parameters of HLA-matched sibling donor (MSD) and haploidentical donor (HID).**

|  | MSD | HID | *P* value |
| --- | --- | --- | --- |
| n (%) | 155 (23.4) | 508 (76.6) |  |
| De novo acute GVHD |  |  | **< 0.001** |
| None | 136 (87.7) | 309 (60.8) |  |
| I | 1 (0.6) | 77 (15.2) |  |
| II | 8 (5.2) | 79 (15.6) |  |
| III | 8 (5.2) | 22 (4.3) |  |
| IV | 2 (1.3) | 21 (4.1) |  |
| Median days (range) from transplant to aGVHD | 27 (8-98) | 20 (3-98) | 0.09 |
| Chronic GVHD |  |  | 0.08 |
| None | 119 (76.8) | 335 (65.7) |  |
| Mild | 28 (18.1) | 130 (25.6) |  |
| Moderate | 6 (3.9) | 3 (6.5) |  |
| Severe | 2 (1.3) | 11 (2.2) |  |
| Median months (range) from transplant to cGVHD | 6.5 (2.1-33.3) | 7.6 (2.1-51) | 0.13 |
| Relapse |  |  |  |
| 1-year | 29 (18.7) | 65 (12.8) | 0.07 |
| 3-year | 37 (23.9) | 90 (17.7) | 0.09 |
| 5-year | 37 (23.9) | 94 (18.5) | 0.14 |
| Relapse location |  |  | 0.55 |
| Bone marrow | 30 (81.1) | 75 (79.8) |  |
| Extramedullary disease | 6 (16.2) | 12 (12.8) |  |
| Both above | 2 (2.7) | 7 (7.4) |  |

Abbreviations: GVHD, graft-versus-host disease.
